# Healthcare with unconditional positive regard: the KICS clinic medical home model for children in out-of-home care

**DOI:** 10.3389/fped.2026.1739614

**Published:** 2026-04-15

**Authors:** Rebecca Orsi-Hunt, Heather Allan, Rachel A. Wilson, Gretchen Domek, Brooks R. Keeshin, Amanda Bird Hoffert Gilmartin

**Affiliations:** 1 Kempe Center for the Prevention and Treatment of Child Abuse and Neglect, Department of Pediatrics, University of Colorado Anschutz Medical Campus, Aurora, CO, United States; 2 Department of Pediatrics, University of Colorado Anschutz Medical Campus, Aurora, CO, United States; 3 Department of Pediatrics, University of Utah, Salt Lake City, UT, United States; 4 Department of Public Health and Caring Science, Child Health and Parenting (CHAP), Uppsala University, Uppsala, Sweden

**Keywords:** foster care, kinship care, medical home model, out-of-home care, pediatric medical home

## Abstract

**Background:**

On any given day in the United States over the past five years 300,000–400,000 children and youth have been in an out-of-home (OOH) placement, typically kinship or foster care. Children living in an OOH setting commonly have health issues beyond those of a general pediatric population. To address the needs of this special population, the Kids in Care Settings (KICS) Clinic was established at Children's Hospital Colorado in May 2023.

**Methods:**

This study provides a comprehensive and descriptive summary of the novel specialty primary care medical home model used at KICS. We use early data from electronic health records to describe the patient population over the clinic's first two years (May 2023 through June 2025), including demographics, placement characteristics and foster and kinship family needs.

**Results:**

Data show that care at KICS is accessible to both foster and kin families, and barriers to keeping appointments are low. KICS provides care across a wide age range and continuous care across placement changes. Care is comprehensive, provided by a multidisciplinary team, and incorporates screening for broader concerns such as educational challenges and substance use. Data show that kinship caregivers have a substantially higher level of psychosocial need and are deeply affected by social determinants of health.

**Conclusions:**

The KICS clinic offers a novel and promising specialty primary care medical home model to deliver care for a diverse population of children and youth living in varying forms of OOH placement.

## 1 Introduction

### 1.1 Background and literature

Foster care in the United States refers to the temporary custody that a child welfare jurisdiction maintains over a child who has been deemed unable to safely remain in their home due to issues of child maltreatment. This temporary placement may occur in any number of settings including family foster care, congregate care, and court-ordered or informal kinship care; for the purposes of this article, we will refer to any of these placements under the umbrella term of “out-of-home” (OOH) care or placement. On any given day in the United States over the past five years between 300,000 and 400,000 children and youth are in an OOH placement (1) with the average length of placement estimated at 22.5 months based on the most recent available data (2). Nearly all children entering OOH care have one or more health care issues at time of entry ranging from birth defects, mental health issues and developmental delays to dental issues; this is to say nothing of the maltreatment or other traumatic experiences that may have resulted in their OOH placement or the upheaval resultant of the removal and placement itself (3, 4).

Generally speaking, children in OOH care experience worse outcomes than their counterparts in the general population in a number of domains, from physical health to mental and behavioral health to educational achievement, mortality, housing insecurity and carceral system involvement. Statistics for youth who “age out” of OOH care without being reunified with their family of origin or being adopted are at particularly high risk of these adverse outcomes, such as increased rates of homelessness, incarceration, and teenaged parenthood (5). Regarding health outcomes specifically, the adverse physical, mental, and behavioral health outcomes for children and youth in OOH care are well documented (e.g., 4, 6–10) and have resulted in the American Academy of Pediatrics (AAP) classifying youth in care as a special health care population. For example, a study by Turney & Wildeman (11), found that children placed in foster care were twice as likely to have learning disabilities, developmental delays, asthma, obesity and speech problems; three times as likely to have an ADHD diagnosis or vision problems; five times as likely to have anxiety, six times as likely to have behavior problems and seven times as likely to have depression, compared to counterparts not placed in care. Further, there is evidence that care needs can differ substantially by the type of out-of-home care that children experience such that children in foster care vs. voluntary kinship care (i.e., without formal child welfare involvement) experience higher rates of prescription medication use, access to mental health services, and reports of abnormal behavior at time of placement (12). These differences themselves can then go on to impact health outcomes; however, the underlying root cause of these differences is unclear.

Much has also been written about the challenges present in meeting these health-related needs and the adverse consequences of failing to meet them (4, 13, 14). Critically, lack of access to health care has been identified as a contributor to the health-related disparities experienced by children and youth experiencing OOH care, including challenges of continuity of care associated with both the initial OOH placement as well as subsequent placement changes. These challenges are often exacerbated by unavailable or incomplete health histories that may be due, in part, to lack of parental consent for sharing of health information as well as a lack of clarity around where crucial information around prior diagnoses, vaccination histories, hospitalizations, etc. may live and who is legally allowed to access them without breaching local confidentiality laws (4, 15).

However, there remains a lack of rigorous evidence around “what works” for healthcare delivery for children and youth in OOH care. Since around 2010, specialized “medical homes” for children in OOH care have been an increasingly cited recommendation (e.g., 16–19); this is likely due in part to the 2008 Fostering Connections to Success and Increasing Adoptions Act's emphasis on prompt medical assessment and care coordination/continuity. The AAP defines medical homes having seven core components, which were introduced via policy statement in 1992 and further operationalized in 2002. They should be: accessible, family-centered, continuous, comprehensive, coordinated, compassionate, and culturally effective (20). The original guidelines were developed with the general pediatric population in mind. However, the AAP formally recommended the pediatric medical home model for children and adolescents in OOH care in its October 2015 policy statement titled “*Health Care Issues for Children and Adolescents in Foster Care and Kinship Care*” (4) in recognition of the unique vulnerability and challenges to receiving accessible, comprehensive, and continuous medical care for this population.

While discussions of medical homes are typically and logically focused on the care needs of children, there are also implications for caregivers related to care access. Kin caregivers, most typically referring to individuals biologically related to the children in their care, such as grandparents, aunts, uncles, and cousins, though sometimes referring to fictive kin such as close family friends or non-relative godparents, are one group of caregivers that has been identified as having unique challenges to accessing services on behalf of the children in their care in addition to high resource needs themselves. This is a necessary consideration when conceptualizing how to deliver healthcare for children in OOH placement due to increased rates of kinship placements in recent years due, in part, to prioritization of kin and family-like placements for children in OOH care legislated in the Family First Prevention Services Act of 2018. Indeed, in federal fiscal year (FFY) 2024, 43% of children entering out-of-home care entered a living arrangement with relative or kin (1); this is a substantial increase from the 27% of children placed in kin settings in FFY 2011 (21). While the literature is clear that kinship caregivers are not a monolithic group, studies suggest that they are often subject to greater difficulty in accessing various caregiving support services (such as caregiver subsidies, parent training, and peer support), direct services, such as healthcare for the children in their care, and experience greater financial stress than their non-kin caregiving counterparts (18, 21, 22).

Despite widespread recognition and recommendation from social service and health care experts around the potential utility of pediatric medical home models in serving this population that faces unique adversities, our team's recent brief review of the extant literature found only four evaluations of such medical home models in the United States (23–26), and these evaluations were often narrowly focused around a limited set of health-related outcomes or costs. Further, comprehensive “rich descriptions” of these models are also lacking. Indeed, in searching both the peer-reviewed literature and gray literature, descriptions of what existing medical home models provide and how only yielded a few results. These included Espeleta et al's (13) description of a primary care clinic housed within a large, Midwestern academic medical center and Garbus's (27) description of the Foster Care Program at the Holyoke Health Center in Holyoke, Massachusetts. This presents a substantial gap in the knowledge base, regarding not only the effectiveness of medical home health care provision for children in OOH care but also simply descriptions of what such care models can look like. Indeed, such descriptions are a necessary precursor for understanding *how* such models may be effective in producing specific outcomes. This study seeks to fill that gap.

### 1.2 Kids in care settings clinic (KICS)

To address health-related challenges for children and youth in OOH care, the Kids in Care Settings (KICS) Clinic was established as a specialty primary care clinic, staffed by a multi-disciplinary team including integrated behavioral healthcare at Children's Hospital Colorado. The clinic saw its first patient in May 2023. The clinic's vision is “Children, youth, and families involved in out-of-home placement leading healthy, resilient lives with empowerment and hope.” The KICS team has developed a comprehensive medical home model based on input from people with lived experience in the child welfare system (including biologic parents whose children have been removed, foster parents, youth with child welfare involvement, and child welfare professionals). This input included the following priorities: enhanced cross-system communication, coordination of care in a single clinic (a one stop shop), access and resources for mental and behavioral healthcare, a trauma-informed approach to care, and a clinic environment of respect with no judgement. The KICS Clinic specifically partners with Arapahoe County Department of Human Services. In Colorado's state-supervised, county-administered child welfare system, Arapahoe is the county providing child welfare services for the geographic area most centrally surrounding Children's Hospital Colorado. Arapahoe County is one of 64 counties in Colorado; it is a large (805 square miles) county in the Denver Metro area that spans 13 cities and towns in both urban and rural areas. The KICS care model is unique in its comprehensive approach to healthcare provision and coordination for children in out-of-home care and the families which provide such care.

### 1.3 Study aim

The current study's aim is to provide a comprehensive and conceptual description of the KICS medical home model, aligned with the American Academy of Pediatrics medical home approach (20). Also, we use early data from electronic health records (EHR) to describe the patient population over the clinic's first two years (May 2023 through June 2025), including demographics, placement characteristics and foster and kinship family social needs of the early patient population at KICS. We use the available data to begin to demonstrate how the medical home model is being operationalized at KICS. To the best of our knowledge, this study will comprise the first model description of a truly comprehensive and trauma-informed medical home model for children and youth in out-of-home placement to appear in the medical literature. This work is a foundational step as we launch a comprehensive and pragmatic evaluation and research program to examine implementation and outcomes for the KICS Clinic.

### 1.4 KICS medical home model description

Using the seven criteria established in the AAP's “medical home” definition as discussed above, we describe the KICS Clinic medical home care model for children who are in an out-of-home placement.

The KICS Clinic makes healthcare **accessible** for children and youth in out-of-home care in several ways. KICS serves children both in foster and kinship placements. KICS accepts Colorado's Medicaid plan and private insurance. This allows provision of care to all children in out-of-come placement (covered by Medicaid) as well as continuation of care for children who have reunified with their biologic family or been adopted (covered by Medicaid or a non-Medicaid insurance plan). KICS appointments are available two days every week, and patients can access the Child Health Clinic (CHC), the general pediatrics clinic, at Children's Hospital Colorado (same physical location) for acute care appointments outside the KICS Clinic regular times (weekdays 8–5 pm and evening clinic hours Monday through Thursday). KICS patients and families can further access nurse triage services 24/7/365. KICS also provides kinship and foster parents with the convenience of allowing their biologic children to be cared for in the same clinic as foster or kin children.

Although some of the above aspects of primary care *may* be available in a typical pediatric primary clinic, KICS increases the accessibility of care in several ways specific to children and youth in out-of-home placement. KICS promptly schedules initial medical assessments for children recently removed or after change of placement, with the KICS social worker reaching out within 48 business hours of receiving a referral to schedule an initial appointment. KICS also facilitates direct access to three types of specialty services commonly used by children in out-of-home care via collaboration with the Children's Hospital Colorado dental clinic (holds appointments for KICS patients one afternoon per week); the eye clinic (holds appointments for KICS patients one morning per month) and the developmental and behavioral pediatrics clinic (hold appointments for KICS patients one morning per week). The eye and dental clinics are co-located with KICS; developmental behavioral pediatrics is close by at the Children's Hospital Colorado main campus. This type of dedicated access to specialty services goes beyond a typical medical home model but is necessary to provide fully accessible care for children in OOH placements. Similarly, a KICS Nurse Complex Care Coordinator (CCC) handles all referrals to Early Intervention for patients under the age of 3. Lastly, families (biologic and foster/kinship) can communicate directly with a KICS team member via cell phone in whatever way the family prefers (e.g., call, text, or email). This increases access to the healthcare team, as the majority of foster and kinship families do not have access to the EHR patient portal typically utilized for direct communication to healthcare teams.

A true medical home model is **family-centered**, which KICS provides in several ways. Although the KICS clinic partners with the child welfare system in Arapahoe County, Colorado, foster parents may bring children in their care under the jurisdiction of another Colorado county to receive care at KICS. Kinship families can also bring any other children residing in the home of the KICS patient to the clinic. These options allow all the children in one family to receive care at a single clinic.

KICS operationalizes family-centered care in several other ways that specifically involve the child's biologic family. The social worker partners with biologic families to obtain their consent for their children to receive medical care beyond routine care. The social worker facilitates biologic parents attending their child's visit—virtually or in person—for any patient for whom the responsible child welfare agency has determined it is safe for such participation to occur. If transportation is a barrier, the social worker will help arrange transportation to the clinic. Biologic families often say that transportation is one of the greatest barriers to attending an appointment. This may be in part because Medicaid only covers a ride for the person bringing the child to the appointment, which is the foster or kin caregiver. Also, Medicaid assisted transportation requires advance scheduling which can be an additional transportation barrier. If a biologic parent was unable to attend the KICS clinic visit, the CCC calls (eligible^1^) families of the child seen in KICS, and if there are multiple biologic parents/caregivers, the CCC attempts to call everyone. The calls are to ensure that families are aware of the appointment, review recommendations from the appointment, and answer any questions the family may have about the appointment. Finally, family-centered care extends beyond the physical clinic when KICS staff members (social worker or CCC, if invited) attend family team meetings associated with the patient's child welfare case to provide relevant health updates and to connect with biologic family members.

The third aspect of a medical home model addressed by KICS is to provide **continuous** healthcare. As mentioned above, KICS will continue to see children after a permanency determination (i.e., adoption, allocation of parental rights) for foster and kinship families and will see children after reunification with biologic families.^2^ KICS has a mechanism to record placement dates and caregiver type and involvement in the EHR, which facilitates continuity of care for the child even when caregivers change across the time of child welfare involvement. Finally, patients consistently see one of two pediatricians for all routine care at KICS. When a child turns 18, KICS will help transition the child to the adolescent medicine clinic (in the same building as KICS) for continuation of care and where the youth can be seen until age 23.

The KICS Clinic care model is **comprehensive**. The clinic sees children and youth ages 0–18 years for initial placement exams, change of placement exams, well-child care, follow ups, and same day sick appointments. To accommodate more complex health needs, KICS has longer appointment times than a typical pediatric clinic (60 min for KICS vs. 30 min at the affiliated CHC). KICS also offers more comprehensive universal screening as compared to a typical pediatric clinic. This includes: psychosocial screening, developmental screening,^3^ autism screening^4^, post-partum depression screening,^5^ traumatic stress screening,^6^ and screening for substance use, anxiety, and depression.^7^

KICS is staffed by a multi-disciplinary team including: two pediatricians (both trained in child abuse pediatrics), a psychologist, a social worker, a nurse complex care coordinator, a community health navigator, a certified child life specialist, a nurse, medical assistants, and in-person Spanish interpreters. KICS integrates mental and behavioral healthcare with physical healthcare by having a psychologist embedded in the clinic and available for any patient visit if needed. In addition to in-clinic access, the psychologist can offer short-term behavioral healthcare to patients and families, working on specific concerns, serving as a bridge to long-term therapy, and coordination with school counselors and individual therapists. To facilitate longer-term comprehensive behavioral care, the social worker and psychologist provide behavioral healthcare navigation, identifying locations that take insurance, are convenient to where families live, are accepting new patients and provide the recommended services.

The KICS clinic conducts staffing meetings to discuss each patient's care with the entire multidisciplinary KICS team a week prior to seeing them in clinic. This helps the team work proactively around consent and coordination with biologic families, ensuring the team has a pre-visit plan if needed that accommodates the needs of the patient and family. Also, to facilitate partnership with the county child welfare agency, select KICS staff meet twice a month in-person at Arapahoe County Department of Human Services to discuss KICS patients and answer medical questions with the case worker, supervisor, and guardian ad litem (GAL). This helps facilitate a positive relationship with child welfare and ensures everyone has the same information and can work in a coordinated fashion on any identified barriers.

The KICS Clinic care model further meets the definition of a pediatric medical home by being highly **coordinated** for children and youth in OOH care. Coordination with family includes an after-visit summary provided to foster, kinship, and biologic parents in attendance. Also, the KICS social worker emails the after-visit summary to the case worker, case worker's supervisor, and GAL after every appointment. KICS works in close coordination with external partners, including the Arapahoe County Department of Human Services, Developmental Pathways (a community center board that is the Early Intervention broker for Arapahoe County), Colorado Access (Medicaid), Children's Hospital Colorado Government Affairs and Medicaid Strategy Teams, Colorado Hospitals Substance Exposed Newborn Quality Improvement Collaborative (CHoSEN QIC), and Arapahoe County's Safe Babies Court Program implemented by Illuminate Colorado. Finally, KICS is partnering with Safe Babies and Arapahoe County DHS on the Courts KICS Partnership Pilot to improve healthcare integration for infants and families impacted by dependency and neglect court cases in Arapahoe County.

The KICS Clinic strives to deliver **compassionate** care. For starters, the KICS team participates in quarterly trauma-informed care training as a team. This helps facilitate understanding of the physiological components of trauma, the importance of relational health, and the young child's response to traumatic events. It also brings awareness of the difficulties that families involved in child welfare face (e.g., court dates, transportation, interaction with caseworkers, challenging attitudes of healthcare providers toward biologic families, relationships between biologic and foster/kinship families) and honoring biologic families' parental rights to consent for care (e.g., vaccines). Additionally, the child life specialist can help children of all ages cope with the stress and uncertainty of acute and chronic illness, injury, trauma, disability, loss, and bereavement during the clinical visit. They use therapeutic play to help patients get ready for exams and procedures and learn about their care in ways that reduce fear, anxiety, and pain. This is particularly important in the KICS population as patients might have had limited contact with healthcare previously or had incidents of maltreatment that make examinations more sensitive. The clinic also attempts to cultivate compassion for county human services colleagues, who are often carrying very large caseloads and where there is not always a natural affinity or understanding for each other's roles. Finally, clinicians are never “assigned” to KICS; each team member chooses to be part of the KICS Clinic and to work with its patient population.

Lastly, the KICS Clinic care model is **culturally effective** for its population of children and youth in care by reaching out often to families individually, as described above. The clinic also provides language interpretation, including in person for Spanish interpreters or video interpreters for other languages. Importantly, in the context of out-of-home care in public child welfare, cultural effectiveness also includes awareness of and supporting the social needs of kinship families. Unlike typical foster families who volunteer to care for children and are trained and licensed by a child welfare agency, kinship families are more likely to struggle with economic and other psychosocial issues. These challenges are assessed at KICS via items on the psychosocial screening tool discussed below.

## 2 Methods

### 2.1 Study design and setting

The study is an observational, descriptive analysis of the patient population at the recently-established Kids in Care Settings (KICS) Clinic at Children's Hospital Colorado. Details regarding the KICS Clinic and a description of its medical home model have been provided in the introduction above. The medical home model description was developed retrospectively based on author team consensus by reviewing clinic protocols, workflows and staffing structures and by incorporating reflections from KICS clinical team members across the first two years of operation.

### 2.2 Participants and inclusion criteria

The study includes all patients cared for at KICS between its opening on May 8, 2023, through June 30, 2025. The patient list was developed and maintained by the KICS Clinic staff during this time period and used to select patients into the study.

### 2.3 Data sources

All study data were extracted from the electronic health records system used by Children's Hospital Colorado and the KICS Clinic. EHR extract tables contained patient-level records, event-level records for KICS Clinic encounters during the study period and information from screeners and flowsheets used in the KICS Clinic. In addition to information typically found in an EHR, the Children's Colorado EHR contains several custom flowsheets for the KICS Clinic documenting information relevant for pediatric patients in OOH care.

### 2.4 Measures

Patients' **age, race, ethnicity and sex** are taken from the electronic health records, typically entered at new patient registration.

A **placement type** variable describes the placement in which the KICS patient lives and is categorized as foster, kin or neither foster/kin. A foster care placement is typically non-relative care and a kinship placement is typically with a member of the child's biologic family or family network. The variable is coded as *foster* if a foster parent or sibling was present at an encounter, *kinship* if a kinship caregiver or sibling was present and *neither* if only an adoptive parent or biologic parent or professional was present. *Neither* characterizes an encounter for the biologic child of a foster parent, a reunified child with biologic parent or a child accompanied by a caseworker or guardian ad litem. Since placement type can change between encounters, we have coded placement type in two ways (1): by the patient's *first-observed* placement type in the EHR and (2) by whether the placement type was *ever-observed* for that patient. For analyses using the *ever-observed* coding, a few children who changed placement types are counted in both foster and kin categories. The **number of placements** over the study time period was determined by counting how many placement dates were present in the EHR for each child.

We extracted data from a **psychosocial screening tool** for caregivers which was developed at the University of Colorado (28); it is also used both at KICS and at the general pediatrics clinic at Children's Hospital Colorado. The caregiver completes this screener at new patient appointments, change of placement appointments and well child checks. The screener is detailed in the Appendix and asks about: barriers to accessing healthcare for child and adult, child's education needs, financial and basic needs, caregiver mental health, household substance use and exposure to threats or violence.

The **Screening to Brief Intervention tool** (29) was used to assess frequency of substance use for older children and youth. The Screening to Brief Intervention (S2BI) is typically administered at initial exams, follow-ups, and well child checks for patients age 11 and older. Patients complete the screener themselves on paper (which is then entered in the electronic health record) and it is reviewed with the KICS provider. The tool screens for multiple substances at frequencies of never, once or twice, monthly, weekly or more.

### 2.5 Analytic approach

The selection of measures described above was guided by the study's aim to use available data from the EHR to describe demographics, placement characteristics and foster and kinship family social needs of the KICS patient population. We organized these descriptive results to align with several of the criteria of the AAP model based on initial team consensus; we have not yet analyzed EHR data that supports every one of the seven criteria. We expect decisions regarding how to operationalize measurement of care delivery within each of the seven AAP domains to evolve as we continue to develop a larger research agenda for KICS. We also expect that data sources will expand beyond the EHR.

From a data analytic perspective, all analyses are descriptive. For all analyses, relevant EHR extract tables were joined to each other using an arbitrary patient identifier to generate counts of patients with a specific child or caregiver characteristic and to calculate percentages as shown in the tables of results. The psychosocial screener is a repeated measure, so most children (88%) had multiple sets of screener data, including some children who could have been patients at Children's Hospital Colorado before they were patients at KICS and consequently had screener data from before KICS opened. Responses from before KICS opened were filtered out. If the screener was completed more than once for a child by the same caregiver type (foster or kin) with the same response, the response is only counted once. Results below show the percent of patients whose caregiver ever responded “yes,” by placement type. All results are for an initial cohort of KICS patients who met the study inclusion criteria and reflect varying lengths of time that each patient has been at KICS.

### 2.6 Ethical considerations

Due to the detailed nature of KICS patient data and the disclosure of KICS' location at Children's Colorado, we have masked the initial patient cohort size and show only percentages throughout the results. This allows us to share more detailed results for patient demographics and psychosocial needs while also minimizing the risk of re-identification that comes from reporting small cell sizes of EHR data. For this reason, total sample size is not disclosed above in “Participants.” This study was determined by the Colorado Multiple Institutional Review Board to be exempt from review (25-0441), due to the exclusive use of secondary EHR data.

## 3 Results

Descriptive results support the second aim of the study, using data from the EHR to describe the patient population over the first two years—including demographics, placement characteristics and foster and kinship family needs. To maintain focus on the KICS Clinic's evolving medical home model, we have organized these descriptive results along five the seven AAP medical home criteria—*accessibility*, *family-centeredness*, *continuity* and *comprehensiveness* of care and *cultural effectiveness* (20).

### 3.1 Demographics

Tables 1, 2 show breakouts by race/ethnicity and sex, and by their first-observed placement type in the electronic health record for the entire study cohort. For comparison, the child population of Colorado is 5% Black and 34% Latiné (30) suggesting that Black and Latiné patients are over-represented in the KICS Clinic patient population. Notable is the high proportion of children identified as Latiné who are in Kinship care at 25% of the KICS patients.

**Table 1:** Race/ethnicity and first observed placement type.

Race/Ethnicity	Foster	Kinship	Neither Foster/Kin	Total
White	20%	13%	–	33%
Black	11%	5%	3%	18%
Hispanic or Latiné	13%	25%	7%	44%
Asian	1%	–	–	1%
Other	1%	–	–	1%
Declined to Respond	2%	2%	–	3%
Total Cohort	47%	44%	9%	100%

**Table 2:** Sex and first observed placement type.

Sex	Foster	Kinship	Neither Foster/Kin	Total
Male	28%	21%	6%	55%
Female	19%	23%	3%	45%
Total Cohort	47%	44%	9%	100%

### 3.2 Accessibility and family-centeredness

Patient and family data indicate that care at KICS is **accessible to both foster and kin families** as shown by the placement type breakouts in Tables 3, 4 below. As shown in Table 3, patients are split almost evenly between foster homes and kinship. **Family-centeredness** is demonstrated by the nine percent of children in the *neither* category at first observation, many of whom are biologic children of foster caregivers, cared for at KICS as part of a family-centered approach. (Neither is also used if the designation in the medical record was missing, than 2% of children). For the study cohort, data are broken out by first observed placement type (Table 3) or by whether a child *ever* experienced a placement type while a patient at KICS (Table 4). Note in Table 4 that 54% children who were ever in a kin placement and 52% children who were ever in a foster placement add to more than 100% due to placement type changes.

**Table 3:** Accessibility—first observed placement type.

Placement (1st observed)	
Foster	47%
Kinship	44%
Neither Foster/Kin	9%
Total Cohort	100%

**Table 4:** Accessibility—ever observed placement type.

Placement (Ever)	
Foster	54%
Kinship	52%
Neither Foster/Kin	23%
Total Cohort	100%

Percents add to over 100% because over the course of care children may move placements or reunify.

Over the prior two decades, there has been an increased focus in the US on using kinship care for OOH placement. In Colorado, this dynamic has resulted in many more children entering kinship placements. The trend implies a need to segment the clinic population by placement type in order to adequately describe it.

Further evidence of accessibility comes from information in the psychosocial screening tool. These results are presented in Table 5. For Table 5 the kinship column shows results for the portion of the cohort who were ever in a kin placement (54% of study cohort). The foster column shows results for the portion of the cohort who were ever in a foster placement (52% of entire cohort). For example, 10% of KICS patients ever in kin care had a kinship caregiver who responded “yes” to “Do you have concerns or problems that make it hard to keep doctor appointments or manage health care?” Among those 10% specific barriers indicated include: childcare, caregiver's job, financial, transportation and work/school stress. Only 3% of KICS patients ever in foster care had a foster caregiver indicate problems with accessibility of care.

**Table 5:** Accessibility of care—barriers to care.

Barriers to care	Kinship	Foster
Problems keeping health appointments or managing child's healthcare	10%	3%
*Childcare*	3%	–
*Caregiver job*	1%	–
*Money/Financial problems*	1%	–
*Transportation*	6%	–
*Work or school stress*	1%	–

Percentages in each column are out of the portion of the KICS cohort who had experienced the specific placement type. For example, 10% of KICS patients who had ever been in kinship care during their time at KICS had a kin caregiver indicate problems keeping health appointments.

Overall, these numbers are low, indicating that most caregivers are able to access care at the KICS Clinic. Facilitating transportation—the most common barrier cited by kinship caregivers—is a major focus of the social worker at the KICS clinic.

### 3.3 Continuity

**Figure 1:**
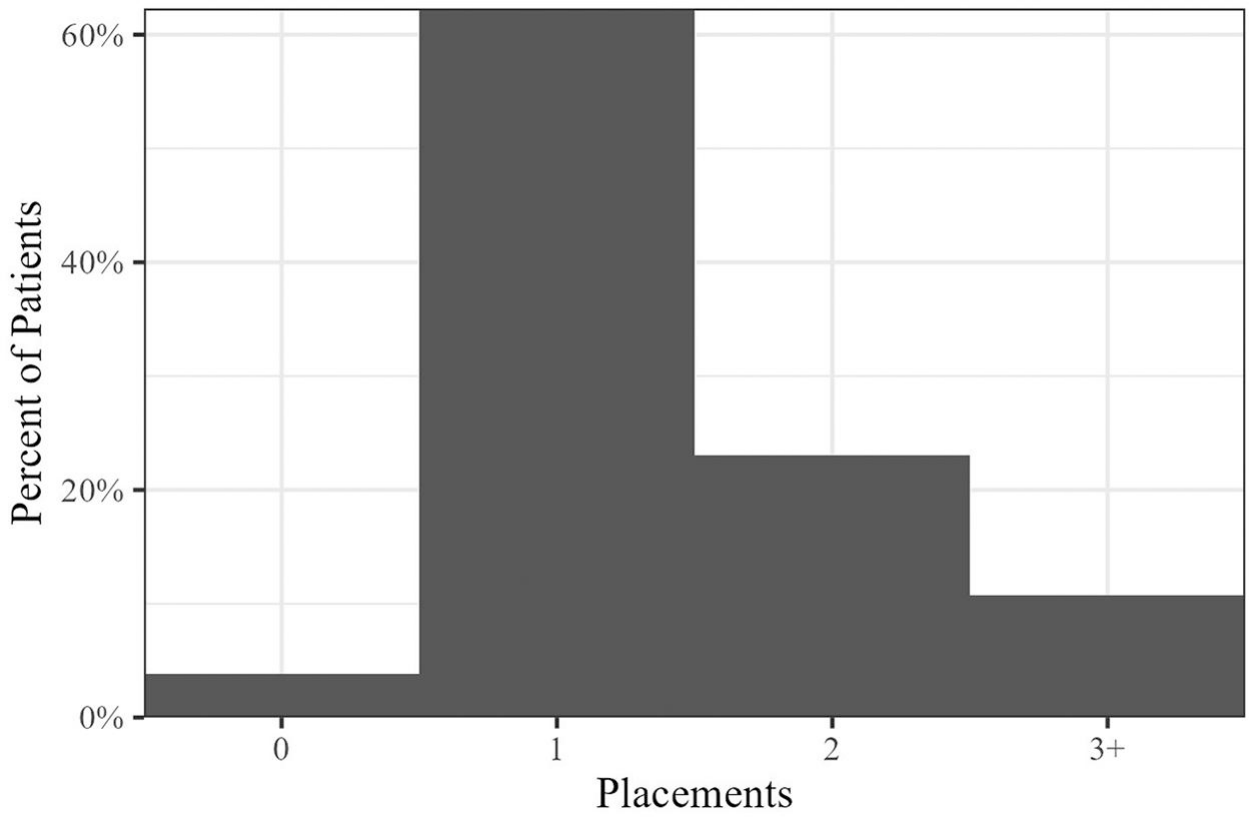
Continuity of care—care across placements.

The data show that care at the KICS clinic is **continuous**; in the context of children in care a key indicator for continuity is whether care persists across placement changes. Figure 1 below shows the distribution of the entire KICS patient cohort by number of placements over time while at KICS; this was determined by counting how many placement dates were present in the EHR for each child. Patients had anywhere between zero and 7 placements while patients at KICS, with 34% having more than one placement during that time.

Placement changes are common for children and youth in OOH care. We expect that maintaining continuity of primary healthcare in spite of placement changes will facilitate substantially better health outcomes over time for KICS patients.

### 3.4 Comprehensiveness

**Figure 2:**
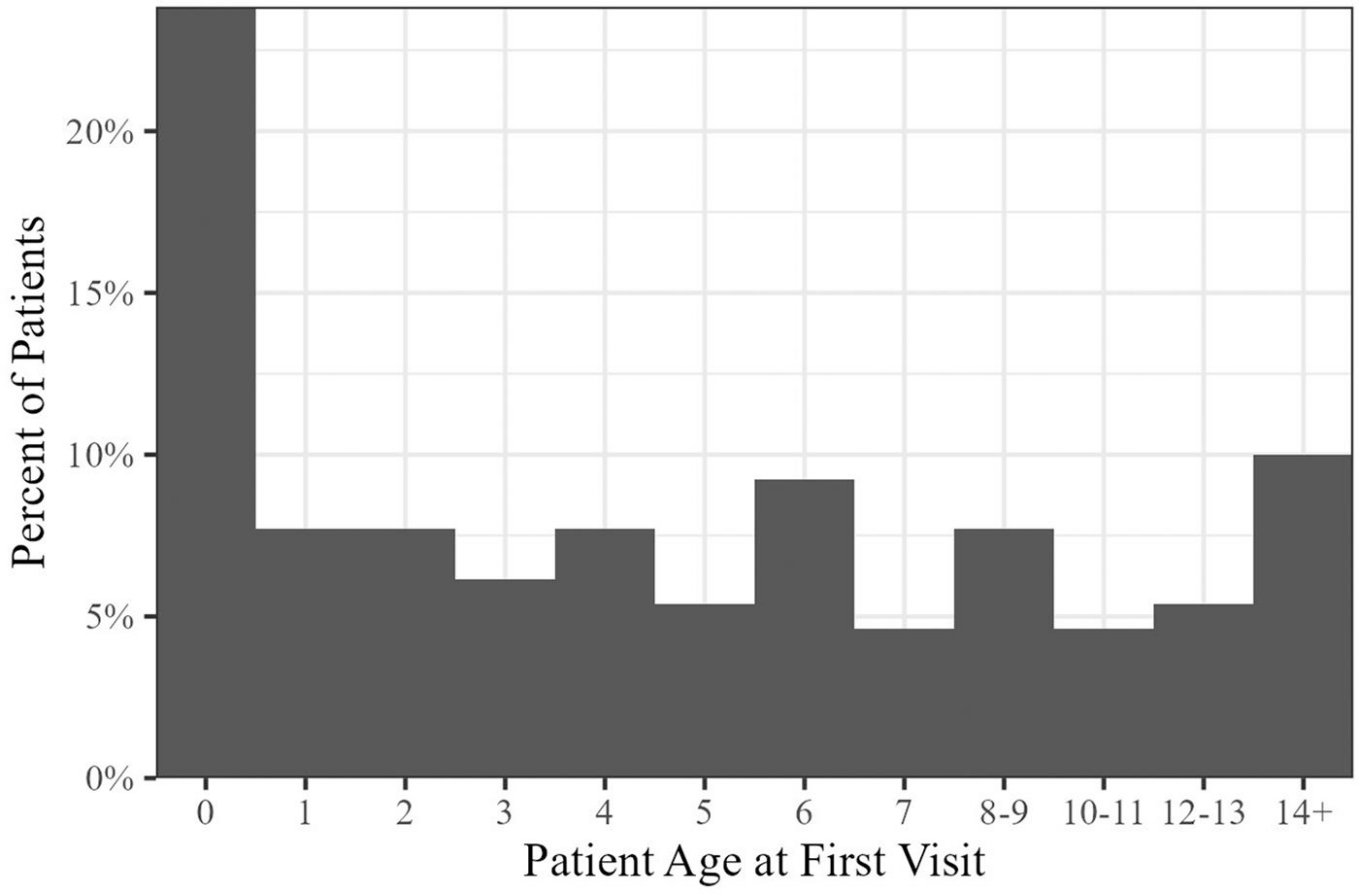
Comprehensiveness of care—patient age at first visit to KICS.

Data show that KICS care is **comprehensive** in several ways. Figure 2 shows a distribution of patient age at first visit to the KICS Clinic for the entire cohort. This shows that KICS is serving patients over a wide age range from birth to adolescence.

In addition, the psychosocial screening tool asks about information beyond direct health needs. An example is educational needs. Table 6 shows results separately for portion of the cohort who were ever in a kin or foster placement. For children in foster placements, 10% of foster caregivers had concerns about educational needs while for children in kin placements only 1% of kinship caregivers indicated such concerns. Specifically, foster caregivers mentioned concerns regarding an individualized education program (IEP) for special education needs and a secondary transition plan which supports the transition out of high school to post-school activities for student with an IEP. KICS providers and staff may engage caregivers and the Department of Human Services around how to address an educational need and what resources may be available.

**Table 6:** Comprehensiveness of care—educational needs.

Educational needs	Kinship	Foster
Concerns about education needs	1%	10%
*Individual Education Program (IEP)*	–	1%
*Secondary Transition Program*	–	1%

Percentages in each column are out of the portion of the KICS cohort who had experienced the specific placement type.

Finally, the Screening to Brief Intervention tool (S2BI) is administered for patients 11 and older to assess substance use within the last year. Table 7 shows results for the portion of the KICS population age 11 and older who were screened for substance use. As shown, experimentation with or use of marijuana is most common.

**Table 7:** Comprehensiveness of Care—Substance Use.

Substance	Never	Once or Twice	Monthly	Weekly or More
Tobacco	55%	15%	15%	–
Alcohol	55%	20%	10%	–
Marijuana	35%	30%	10%	10%
Vape	55%	5%	–	–
Prescription Meds^a^	60%	10%	–	5%
Illegal Drugs	80%	15%	–	–
Inhalants	70%	5%	–	–
Synthetics^b^	70%	–	–	5%

^a^ Prescription drugs that were not prescribed.

^b^ Herbs or synthetic drugs (such as salvia, “K2” or bath salts).

Percentages are out of KICS patients age 11+ with an S2BI response.

Of note, the S2BI is validated to a youngest age of 12 years. However, the KICS clinic has special approval from the Children's Hospital Colorado Questionnaire Governance Committee to use it for patients 11 years and older, given the high-risk nature of our population for substance use concerns and the additional supports the clinic can provide to patients in completing the screening tool. Also, as described below, caregiving families are screened for substance use.

### 3.5 Cultural effectiveness

Culturally effective care includes an understanding of a care-giving family's social context. The previously-described psychosocial screening tool covers many aspects of social needs. Table 8 shows these results separately for portions of the cohort who were ever in a kin or foster placement. Twenty-five percent of KICS kin caregivers report “stress about making ends meet during the last 3 months” while this type of stress is reported by only 6% of foster caregivers. More specifically, 19% of kin caregivers report stress related to gas/transportation, 19% with respect to paying bills and 15% report stress related to paying the rent or mortgage. This contrasts with no foster caregivers reporting stress around gas/transportation or rent/mortgage needs and only 3% reporting stress related to paying bills. Twenty-five percent of kinship care families had an instance in the last 3 months when food in the home did not last, and they didn't have money to get more. Twenty-one percent want to learn about benefits to assist with housing, healthcare and other basic needs. In contrast, only 1% of foster caregivers report an instance of food running out without the ability to purchase more and only 6% express interest in learning about economic and health benefit programs. Finally, 13% of kin caregivers vs. 4% of foster caregivers reported concern about housing, including the possibility of becoming homeless.

**Table 8:** Culturally Effective Care.

Family needs	Kinship	Foster
Stress about making ends meet during last 3 months	25%	6%
*Childcare*	1%	–
*Diapers*	9%	–
*Gas/Transportation*	19%	–
*Paying bills*	19%	3%
*Rent/Mortgage*	15%	–
Worry that food would run out before you had money to buy more (in last 3 months)	31%	4%
Did food ever not last and you didn't have money to get more (in last 3 months)	25%	1%
Would like to learn about benefit programs that may help with basic living costs	21%	6%
*Child Care Assistance Program (CCAP)*	1%	1%
*Food Stamps (SNAP)*	7%	1%
*Medicaid/Child Health Plan Plus (CHP+)*	9%	–
*Medicaid Waivers*	1%	–
*Medical legal partnership*	1%	–
*Temporary Assistance for Needy Families (TANF)*	3%	–
*Unemployment Insurance*	1%	–
*WIC*	3%	1%
Concerns about housing or becoming homeless	13%	4%
Caregiver needs help finding a doctor or clinic for themselves	15%	3%
Want to talk to someone about feeling alone/needing someone to rely on for problems	3%	4%
Problem in the home with alcohol or marijuana	6%	1%
Someone in the home uses medicine not as prescribed or other drugs	–	1%
Feel sad, hopeless or anxious a lot of the time	13%	1%
Caregiver or child been threatened, hit, slapped or touched in unwanted way in last year	3%	1%

Percentages in each column are out of the portion of the KICS cohort who had experienced the specific placement type.

Regarding physical and behavioral health needs, 15% of KICS patients ever in kinship care had a kin caregiver report that the caregiver needed help finding a doctor or clinic for themselves, compared to 3% of foster caregivers. Six percent of kin caregivers reported that someone in the home had a problem with alcohol or marijuana (vs. 1% of foster caregivers reporting this challenge); one percent of foster families reported that someone in the home had a problem with prescription or other drugs. And 13% of kin caregivers reported feeling “sad, hopeless or anxious a lot of the time” while only 1% of foster caregivers reported such feelings.

Finally, the screener also asks about safety concerns (threats, physical violence, unwanted touch). Caregivers are asked to respond to this question regarding their current situation and current children placed with them. However, the question can be open to interpretation, and some caregivers do answer it based on past events. Clinic providers and staff ask clarifying questions about any positive response to assess whether there is an active safety issue that needs to be addressed. Overall, 3% of kin caregivers and 1% of foster caregivers responded affirmatively to this question.

Data presented in Table 8 show that a variety of psychosocial needs are much more prevalent among kinship families than among foster families. This includes caregiver access to healthcare, anxiety/depression symptoms, basic material and economic needs, worry over benefit programs, family substance use and safety. We discuss implications of these results below in relation to care delivered at KICS.

## 4 Discussion

The study's goal is to establish in the literature a rich description of a novel medical home model for children in out-of-home care and to provide a foundation for a pragmatic evaluation and research agenda examining model implementation and patient and family outcomes at KICS. We have provided a comprehensive summary of the KICS medical home model and used readily available data from the electronic health records system at Children's Hospital Colorado to describe the patient demographics, placement characteristics and foster and kinship family social needs.

The initial findings highlight four major themes: an even distribution of foster and kinship families served; a substantial proportion (34%) of patients receiving care across one or more placement changes; a wide age range of children served; and a notable disparity in psychosocial needs, with foster and kinship families experiencing different levels of challenges related to financial stability, transportation, housing, and food security. These findings are consistent with known challenges of high placement turnover in this population, higher psychosocial needs for kinship families (even prior to accepting placement of a child into their home), and the importance of the trauma informed approach of serving family systems and providing comprehensive supports inside of a bio-psycho-social model that includes psychosocial needs.

Findings from the study highlight several important implications for pediatric practice and child welfare policy for children in out-of-home (OOH) care. There is a need for integrated, trauma-informed, and coordinated pediatric care for children in out-of-home (OOH) placements. A comprehensive “one-stop shop” approach—closely aligned with child welfare systems—can improve outcomes by streamlining specialty care, enhancing system navigation, and providing caregivers with targeted psychoeducation on trauma and child development. However, foster and kinship families present distinct clinical and psychosocial needs, requiring intentional differentiation in service delivery and supports, particularly in addressing social determinants of health.

Policy efforts are critical to sustaining this model. Flexible funding streams and cross-sector partnerships are needed to address upstream social and economic factors that influence placement stability and child health. These demands highlight the need for multidisciplinary teams and reimbursement structures that account for care coordination and increased administrative time. In addition, policies and clinical guidelines that enable appropriate information sharing, clarify consent processes, and promote caregiver and parent engagement are essential to operationalizing inclusive, coordinated care. Overall, effective pediatric care for children in OOH settings requires resource-intensive, cross-sector models that are responsive to placement type and grounded in both clinical and social context.

As kinship placements continue to increase nationally, pediatric systems must be prepared to address the greater prevalence and complexity of social determinants of health (SDOH) challenges in kinship care. Kinship caregivers often face financial strain, housing instability, childcare challenges, and transportation barriers, compounded by complex relational dynamics with biological parents and ongoing involvement with the child welfare system. Effective care models therefore require not only screening for SDOH, but also active care coordination, flexible funding mechanisms, and staff capacity to assist families in navigating and accessing resources.

The KICS model also underscores the importance of inclusive care practices that intentionally involve foster caregivers, kinship caregivers, and biologic parents. Inclusion of biologic parents during OOH placement supports skill-building and engagement with the healthcare system prior to reunification, reducing disruptions when children return to parental care. This approach recognizes the critical role of biologic parents' knowledge and relationships in supporting children's health, even during periods of placement, as well as centering the importance of relationships, furthermore it aligns with broader child welfare goals of reunification and family preservation.

From a workforce and systems perspective, implementation of comprehensive OOH-focused pediatric models requires greater staffing resources than traditional pediatric clinics. Additional administrative and clinical time is needed for documentation, family outreach, coordination with specialists, communication outside of electronic health record portals, disclosures, and collaboration with child welfare professionals and legal representatives. Future evaluations that map team member roles and time utilization will be essential to demonstrate the value and cost-effectiveness of these models and to inform sustainable funding and staffing policies.

Arapahoe County Department of Human Services reached out to families twice to get feedback regarding their experiences at KICS, both at clinic opening and 18 months later at the end of 2024. This feedback was overwhelmingly positive with families suggesting that KICS was filling a need in healthcare where such a resource did not previously exist. Additionally, feedback has been overwhelmingly positive from child welfare workers and leaders. Given the importance of understanding what families find most helpful and where growth is needed, KICS is conducting a separate, qualitative study on the experiences of foster, kinship, and biological families. Furthermore, numerous foster families have requested to bring children subsequently placed with them to KICS and numerous biologic parents have continued care in the KICS clinic after reunification. These early findings underscore both the urgent need for comprehensive healthcare for children in out-of-home placements and the promise of a comprehensive medical home model. They suggest that meaningful improvements in care delivery will require integrated, inclusive, and resource-intensive approaches that are responsive to placement type, attentive to social determinants of health, and closely aligned with child welfare systems.

### 4.1 Limitations

There are inevitable limitations to this study, given the novelty of the specialty medical home model. First, the sample size is small and as described in section 3.6, cannot be fully disclosed. This issue will resolve itself over time as additional iterations of an evaluation include more patients. The KICS Clinic is a single-site implementation, so it is not yet possible to understand whether and how results might transfer or generalize to other sites and how results are related to specific aspects of the medical home model. The study relies on early-stage, EHR data in an effort to reduce patient and provider and staff data collection burden, prior to undertaking more comprehensive evaluation; early and available data did not support analyses along every one of the seven AAP criteria. Finally, the study lacks any comparison group.

### 4.2 Future work and conclusion

There is a great deal of work ahead of us. Future evaluations will expand the present focus on psychosocial needs to include quantitative evaluations of physical, developmental, and service utilization outcomes. Possible analyses would include immunization status and completion of catch-up schedules; adherence to recommended well-child healthcare; dental evaluations and unmet restorative needs; referrals to specialty medical services; demonstration of use of multidisciplinary team members; utilization of home-based therapies (occupational, physical, and speech therapy); early intervention referrals; and standardized developmental assessments. A sequential mixed methods approach would be appropriate to explain the quantitative results based on EHR data and better understand why specific results are observed. Mixed methods data collection could take place with both families served and professional partners who interface with KICS. Finally, future work will begin to explore comparative evaluation between KICS and a broader pediatric Medicaid-insured population.

In summary, the KICS clinic demonstrates the feasibility and broader relevance of a multidisciplinary medical home model for children in out-of-home care. By serving a diverse, high-risk population across developmental stages and placement types, the model effectively addresses unmet health needs, engages families, and is valued by caregivers and child welfare partners—supporting its potential for replication and wider implementation.

## Data Availability

The data analyzed in this study are subject to the following restrictions: Electronic health records data from the KICS Clinic are not permissible to be shared. Requests to access these datasets should be directed to rebecca.orsi-hunt@cuanschutz.edu.

## Footnotes

^1^Ineligible to receive call: Parental rights have been allocated to another caregiver, parental rights have been terminated, care rendered was for a teen and is confidential (i.e. sexual health, mental health), parent unable to be located, Safe Haven relinquishment, or parent incarcerated (KICS will attempt to contact lawyer if known).

^2^Because the KICS clinic is still new, the timeline for care continuation is not entirely determined yet, but it will be for a minimum of 6-12 months.

^3^Ages and Stages Questionnaire (ASQ), a development screen administered at well child checks between 2 and 60 months.

^4^Modified Checklist for Autism in Toddlers, Revised (MCHAT-R), administered at well child checks at 18, 24, and 30 months.

^5^EPDS at well child checks from 0-6 months, compared to CHC 0-4 months.

^6^Pediatric Traumatic Stress Care Process Model (PTS-CPM); not typical for a pediatric primary care clinic. PTS-CPM is caregiver report 5-10 years of age and self-report 11 years and older.

^7^Screening to Brief Intervention (S2BI), General Anxiety Disorder-7 (GAD7) and Patient Health Questionaire-9 (PHQ9), respectively, for children and adolescents ages 11 years and older.

## **Appendix**—Psychosocial Screener Questions

- Do you need help finding a doctor or clinic for yourself?
- Do you have concerns or problems that make it hard to keep doctor appointments or manage health care?
  - If yes: What concerns and problems do you have?
- In the last 3 months, have you felt stressed about making ends meet?
  - If yes: What things make you stressed about making ends meet?
- Would you like to learn about benefit programs that may help you with food, housing, health care, and other basic living costs?
  - If yes: Which of the following would you like to learn more about?
- In the last 3 months, have you worried that your food would run out before you had money to buy more?
- In the last 3 months, did your food ever not last and you didn't have money to get more?
- Do you have concerns about education needs?
  - If yes: Does your child have any of the following?
- Do you have concerns about your housing or becoming homeless?
- Do you want to talk to someone about feeling alone or needing someone to rely on when you have problems?
- Does anyone in your home have a problem with alcohol or marijuana?
- Does anyone in your home use medicine not prescribed to you, or any other type of drugs (such as cocaine, heroin or meth)?
- In the past year, has anyone threatened, hit, slapped or touched you or your child in an unwanted way?
- Do you feel sad, hopeless, or anxious a lot of the time?
  - If yes: Have you had recent thoughts of harming yourself or others?
